# Immunogenicity of an Electron Beam Inactivated *Rhodococcus equi* Vaccine in Neonatal Foals

**DOI:** 10.1371/journal.pone.0105367

**Published:** 2014-08-25

**Authors:** Angela I. Bordin, Suresh D. Pillai, Courtney Brake, Kaytee B. Bagley, Jessica R. Bourquin, Michelle Coleman, Fabiano N. Oliveira, Waithaka Mwangi, David N. McMurray, Charles C. Love, Maria Julia B. Felippe, Noah D. Cohen

**Affiliations:** 1 Equine Infectious Disease Laboratory, Department of Large Animal Clinical Sciences, College of Veterinary Medicine and Biomedical Sciences, Texas A&M University, College Station, Texas, United States of America; 2 National Center for Electron Beam Research and Departments of Poultry Science and Nutrition and Food Science, Texas A&M University, College Station, Texas, United States of America; 3 Antech Diagnostics, College Station, Texas, United States of America; 4 Department of Veterinary Pathobiology, College of Veterinary Medicine and Biomedical Sciences, Texas A&M University, College Station, Texas, United States of America; 5 Department of Microbial Pathogenesis and Immunology, Texas A&M Health Science Center, Texas A&M University, College Station, Texas, United States of America; 6 Department of Large Animal Clinical Sciences, College of Veterinary Medicine and Biomedical Sciences, Texas A&M University, College Station, Texas, United States of America; 7 Department of Clinical Sciences, College of Veterinary Medicine, Cornell University, Ithaca, New York, United States of America; East Carolina University School of Medicine, United States of America

## Abstract

*Rhodococcus equi* is an important pathogen of foals that causes severe pneumonia. To date, there is no licensed vaccine effective against *R. equi* pneumonia of foals. The objectives of our study were to develop an electron beam (eBeam) inactivated vaccine against *R. equi* and evaluate its immunogenicity. A dose of eBeam irradiation that inactivated replication of *R. equi* while maintaining outer cell wall integrity was identified. Enteral administration of eBeam inactivated *R. equi* increased interferon-γ production by peripheral blood mononuclear cells in response to stimulation with virulent *R. equi* and generated naso-pharyngeal *R. equi*-specific IgA in newborn foals. Our results indicate that eBeam irradiated *R. equi* administered enterally produce cell-mediated and upper respiratory mucosal immune responses, in the face of passively transferred maternal antibodies, similar to those produced in response to enteral administration of live organisms (a strategy which previously has been documented to protect foals against intrabronchial infection with virulent *R. equi*). No evidence of adverse effects was noted among vaccinated foals.

## Introduction

*Rhodococcus equi* is a facultative intracellular pathogen recognized clinically as a leading cause of severe pneumonia in foals [1]–[4]. To date, an efficacious vaccine against *R. equi* for foals is lacking and there is no approved vaccine for foals against *R. equi* in North America. Although a variety of strategies have been evaluated for vaccination against *R. equi* (including immunization of mares [5]–[9], inactivated *R. equi* administered parenterally to foals or mice [7], [10], sub-unit vaccines [8], [9], [11], DNA vaccines [12], [13], and live, mutant vaccines [14], [15]), oral administration of live, virulent *R. equi* is the only vaccination strategy that has been demonstrated repeatedly to protect foals against experimental intrabronchial challenge with virulent *R. equi* [16]–[18]. However, the administration of live, virulent organisms is not considered an acceptable strategy for vaccination of foals at horse breeding farms because of concerns for environmental dissemination and the potential to cause disease in some foals.

Inactivated bacteria and viruses can elicit protective immune responses against systemic infections, including those of the respiratory tract [19]–[22]. Electron beam (eBeam) irradiation is a technology for microbial inactivation that is currently used for sterilization and pasteurization [23]–[26]. Electron beam irradiation at appropriate doses can be used to inactivate large volumes of microbial cultures or to sterilize materials such as medical devices [27]–[29], and has advantages relative to heat or formalin inactivation. Inactivation with either heat or formalin is known to denature proteins, including immunogenic epitopes on the cell surface [30], [31]. More importantly, formalin is widely recognized as resulting in incomplete inactivation of organisms, and has been associated with vaccine-associated disease resulting from inadequate inactivation [32]. Thus, there is need for a reliable method of microbial inactivation that will retain the bacterial cell structure as similar as possible to a live organism for use in producing vaccines. We therefore identified a dose of eBeam irradiation that would inhibit bacterial replication while maintaining outer membrane integrity of *R. equi*, and examined the immunogenicity of *R. equi* inactivated accordingly when administered enterally to newborn foals.

## Material and Methods

### Ethics statement

All procedures for this study were reviewed and approved by the Texas A&M University Institutional Animal Care and Use Committee (protocol number AUP# 2011-124) and the Texas A&M University Institutional Biosafety Committee (permit number 20110183-Cohen). The foals used in this study are owned by Texas A&M University, and permission for their use was provided in compliance with the Institutional Animal Care and Use Committee procedures.

### Preparation of bacteria and electron beam irradiation

*Rhodococcus equi* strain EIDL 5-331 (a virulent isolate from a Texas foal) was used for this study. One colony-forming unit (CFU) was inoculated into 50 ml of brain-heart infusion (BHI) broth and shaken for 24 h at 37°C, sub-cultured in 1000 ml of BHI broth and shaken for 24 h at 37°C. The bacterial suspension was centrifuged at 3400×g (5810R, Eppendorf AG, Hamburg, Germany) for 20 min at 4°C, the supernatant discarded, and the pellets washed with 100 ml of phosphate-buffered saline (PBS), using the same centrifugation protocol. The supernatant was discarded, the bacteria were resuspended in sterile 0.9% NaCl solution, and the concentration of bacteria was determined spectrophotometrically (Genesys 20, Thermo Scientific, Waltham, MA, USA). For eBeam dose identification experiment, 25 ml of bacterial suspensions of either approximately 1×10^8^ (concentration 1) or 1×10^9^ CFU/ml (concentration 2) were double-bagged in heat-sealed sacs with no headspace, sealed inside a 95-kPa transport bag (Therapak, Duarte, CA, USA), and exposed to irradiation doses ranging from 0 to 7 kGy (in integer-unit doses) using a 10-MeV, 18-kW linear accelerator. Alanine dosimeters were used to verify the delivered eBeam dose. The interaction of ionizing radiation with alanine releases free radicals [33], which were measured by electron paramagnetic spin spectroscopy (E-scan, Bruker BioSpin, Corp., Billerica, MA, USA). Twenty-five ml of non-irradiated bacteria were inactivated for 30 min in a water bath at 85°C, and were used as the heat-inactivated negative control. After irradiation, quantitative culture was performed to determine the concentration of replicating *R. equi* in each irradiated sample, and to calculate the D_10_-value, the dose required for 90% reduction of the initial population [40]. Experiments were conducted in triplicates, performed on 3 different days. For vaccine preparations administered to foals, eBeam irradiated *R. equi* were cultured on days 1, 3, 5, 7, and 14 post-irradiation to confirm absence of bacterial replication.

### Cell wall integrity of irradiated *R. equi*

The immunogenic proteins of *R*. *equi* are expressed on the surface of the bacterium [34]; therefore, maintaining cell wall integrity is important for retaining the immunogenicity of a whole organism. Bacteria were grown as described above, and were eBeam irradiated at the minimum dose that effectively inactivated all microorganisms for the bacterial concentration; live and heat-inactivated *R. equi* were prepared as positive and negative controls, respectively. Samples were kept at 4°C for 12 h, and 1, 2, and 4 weeks after either irradiation or heat-inactivation. Two methods were used to determine whether the bacterial cell wall was intact. The first was a fluorescence-based assay (LIVE/DEAD BacLight bacterial viability kit, Molecular Probes, Inc., Eugene, OR, USA), which utilizes a mixture of SYTO 9 green-fluorescent nucleic acid stain that stains all bacteria, and propidium iodide that only penetrates damaged membranes [35], used according to the manufacturer's instructions. Briefly, bacterial samples were treated with either PBS (does not damage the integrity of the cell wall) or 70% isopropyl alcohol (should cause damage to the cell wall). Then, a series of tubes containing a mixture with percentages of PBS treated:alcohol treated bacteria (0∶100, 10∶90, 50;50, 90∶10, 100∶0) were prepared. Samples were transferred to a 96-well flat-bottom microplate and mixed with staining solution. Fluorescence of both SYTO 9 green and propidium iodide were measured in each well with excitation wavelength at 485 and 530 nm, respectively, using a microplate reader (Synergy 2, Biotek, Winooski, VT, USA). A ratio of green/red fluorescence was calculated (Gen5, Biotek, Winooski, VT, USA) and plotted against the percentage of PBS treated:alcohol treated bacteria. The second method was transmission electron microscopy (TEM) of irradiated samples, heat-inactivated, and live *R. equi* at 12 h, and 1, 2, or 4 weeks after processing. Bacterial cells were fixed in 2% glutaraldehyde, 3% formaldehyde in 0.1 M sodium cacodylate buffer, then post-fixed with 1% osmium tetroxide and 0.5% potassium ferrocyanide, dehydrated in an ascending alcohol series, and embedded in epoxy resin. Ultrathin sections of the cells were examined with an FEI Morgagni 268 transmission electron microscope at an accelerating voltage of 80 kV. Digital images were acquired with a MegaView III camera operated with iTEM software (Olympus Soft Imaging Systems, Germany), and subsequently post-processed with Adobe Photoshop.

### Study animals

Thirty-four healthy Quarter Horse foals and their respective dams were used for this study. All foals had age-appropriate results of complete blood count on day 2 of life, and had adequate transfer of passive immunity as assessed by a commercially-available qualitative immunoassay for serum concentration of total IgG (SNAP test; IDEXX, Inc., Westbrook, ME, USA). All foals were monitored daily by technical staff and twice weekly by a veterinarian, and remained in good health without clinical signs of disease throughout the study. Individual foals were randomly assigned to the following groups: 1) **EBRE 1** group (n = 9) which received 2×10^10^ CFUs of *R. equi* inactivated by 4 kGy of eBeam radiation in 100 ml of saline adjuvanted with 100 µg of cholera toxin B subunit (CTB, List Biological Laboratories, Campbell, CA, USA); 2) **EBRE 2** group (n = 10) which received 1×10^11^ CFUs of *R. equi* inactivated by 5 kGy of eBeam radiation in 100 ml of saline adjuvanted with 100 µg of CTB; 3) **Saline** (negative) control group (n = 9) which received 100 ml of saline adjuvanted with 100 µg of CTB; and, 4) **LVRE** (positive) control group (n = 6) which received 1×10^10^ CFUs of live *R. equi* in 100 ml of saline. All treatments were administered enterally with a nasogastric tube on days 2, 9, 16, and 23 of life. Physiological saline (NaCl 0.9%) was used as a diluent for eBeam vaccines, live bacteria, and the negative control.

### Sample collection

Samples were collected from foals on day 2 (prior to vaccination) and on day 32 of life. For the broncho-alveolar lavage (BAL) procedure, a 3-meter endoscope disinfected with glutaraldehyde prior to use was passed via the nose into the lungs, until the tube became gently lodged in a bronchus. Sterile saline (30 ml) was instilled into the lung via the endoscope's infusion channel, followed by 20 ml of air to flush, and immediately aspirated to recover at least 15 ml of fluid.

Naso-pharyngeal samples were collected by inserting a 16-inch cotton swab pre-moistened with 3 ml of sterile saline in the nasal ventral meatus. The naso-pharyngeal area was swabbed, the liquid was manually squeezed from the swab using a 35-ml syringe into a tube, and samples were frozen at −80°C until assayed.

Blood was collected from a jugular vein into tubes: 5 ml of blood were collected into a tube without anticoagulant and centrifuged at 3000×g for 5 min to harvest serum, which was separated and frozen at −80°C until assayed, and 16 ml of blood was collected into tubes with sodium heparin as an anticoagulant for isolation of peripheral blood mononuclear cells (PBMCs).

Mammary secretions and serum from the foals' respective dams were collected on day 2 postpartum (PP) for assessment of maternal antibodies. Mares and foals are naturally exposed to *R. equi* from the environment [36]; therefore, a considerable level of antibody response was expected from the tested mares and foals from natural exposure.

### Cell-mediated immune response

The cell-mediated immune (CMI) response to vaccination was assessed by interferon- γ (IFN-γ) production by PBMCs following specific stimulation with eBeam inactivated *R. equi*. PBMCs were isolated using Ficoll-Paque gradient separation (GE Healthcare, Piscataway, NJ, USA) and carbonyl iron (Sigma-Aldrich, St. Louis, MO, USA), resuspended in RPMI-1640 media (Gibco, Life Technologies, Grand Island, NY, USA) with 15% fetal bovine serum (Gibco, Life Technologies, Grand Island, NY, USA) and 1.5% penicillin-streptomycin (Gibco, Life Technologies, Grand Island, NY, USA), and cultured for 48 h at 37°C with 5% CO_2_ with either media only, the mitogen ConA (positive control; 5 µg/ml, Sigma-Aldrich, St. Louis, MO, USA), or eBeam inactivated *R. equi* (multiplicity of infection of 10). After 48 h, supernatants from each group were harvested, centrifuged at 300×g, and frozen at −80°C until examined for IFN-γ production using an equine IFN-γ enzyme linked immunosorbent assay (ELISA) kit (Mabtech Inc., Mariemont, Ohio, USA) according to manufacturer's instructions. Optical densities (OD) were determined using a microplate reader Synergy 2 (Biotek, Winooski, VT, USA), and standard curves were generated and IFN-γ concentrations in each sample were calculated for each isotype using the software Gen 5 (Biotek, Winooski, VT, USA).

### Mucosal and systemic humoral immune responses

Mucosal humoral immune responses were assessed by quantifying total and *R. equi*-specific IgA and IgG isotypes IgG_1_, IgG_3/5_, and IgG_4/7_ in BAL fluid, and total and *R. equi*-specific IgA in naso-pharyngeal swab eluates. Systemic humoral response was assessed among foals by quantifying serum concentrations of total and *R. equi*-specific IgA and IgG isotypes (IgG_1_, IgG_4/7_, IgG_3/5_).

Concentrations of total IgA and IgG isotypes (IgG_1_, IgG_3/5_, and IgG_4/7_) were determined by ELISA using a commercial kit (Bethyl Laboratories, Montgomery, TX, USA) according to manufacturer's instructions. Reference serum (Bethyl Laboratories, Montgomery, TX, USA) was added for the positive controls and to establish standard curves, and dilution buffer was used as blank. Optical densities were determined by using a microplate reader. Standard curves were generated and immunoglobulin concentrations in each sample were calculated for each isotype using the software Gen 5.

For determination of *R. equi-*specific IgA and IgG isotypes we used a protocol described previously [37]. Briefly, ELISA plates (Maxisorp, Nalge Nunc International, Rochester, NY) were coated with 2.5 µg/ml of *R. equi* antigen diluted in coating buffer (Carbonate-bicarbonate buffer, Sigma-Aldrich, St. Louis, MO) overnight at 4°C. The protocol for preparation of *R. equi* antigen has been described previously [38], except *R. equi* strain 5–331 was used in this study. Plates were washed five times with Tris-buffered saline (TBS) with 0.005% Tween 20, blocked with 200 µl TBS 1% bovine serum albumin for 30 min at room temperature (RT), and washed again. Two-fold serial dilutions of serum and mammary secretions samples from study foals, positive control *R. equi* hyperimmune plasma (Mg Biologics, Ames, IA), and negative control fetal horse serum (Biowest, Miami, FL, USA) were added in duplicates to the wells and incubated for 60 min at 22°C. Both BAL fluid and naso-pharyngeal (NP) swab eluates were used undiluted. After another washing, goat anti-horse IgA, IgG_1_, or IgG_3/5_ peroxidase conjugated, or sheep anti-horse IgG_4/7_ peroxidase conjugated (Bethyl Laboratories, Montgomery, TX) were added to the wells and incubated for 60 min at RT. Plates were washed again, and TMB One Component HRP Microwell Substrate (Bethyl Laboratories, Montgomery, TX) was added to the wells and incubated for 15 min at RT in the dark. The reaction was stopped by adding sulfuric acid solution to the wells. Optical densities were determined by using a microplate reader. Relative quantities for day 2 and day 32 samples were obtained by using the following formula:
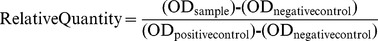



The ratio of the relative quantities on day 32 to the relative quantities on day 2 was used to describe the relative increase/decrease of antibodies following vaccination.

### Data Analysis

Analysis of growth curves and cell wall integrity fluorescence data were performed using linear mixed-effects models with experimental replicates modeled as random effects [39]. The D_10_-value was calculated from the negative inverse of the slope from the linear mixed-effects regression of the irradiation dose on the logarithm_10_ of the microbial population. [40]. Transmission electron microscopy data were descriptive only.

Foal data were analyzed using the ratio of relative quantities on day 32 to relative quantities on day 2 of life (baseline) of immunoglobulins or IFN-γ concentrations, or the proportion of foals that had an increase in these relative quantities. When appropriate, data were log_10_-transformed to ensure they met the assumptions underlying the modeling strategy.

Ratio data were analyzed using a generalized linear model with concentrations as the outcome variable and study group as the independent variable of interest. Model fit was assessed by examining diagnostic plots of residuals. Post-hoc pair-wise comparisons among groups were made using the method of Sidak [41]. Proportions of foals with increased immunoglobulin isotypes were compared among groups using Fisher's exact test.

Association (correlation) between immunoglobulin concentration in mare serum and mammary secretions immunoglobulins, and mare mammary secretions and NP swab eluates IgA were made using linear regression analysis. Comparisons of immunoglobulin concentrations among treatment groups were performed using Kruskal-Wallis testing. All analyses were conducted using S-PLUS (Version 8.0; Insightful, Inc.) and R (Version 2.12.1; R Statistical Project) and a significance level of P<0.05.

## Results

### Effects of eBeam irradiation on *R. equi*

**Figure 1:**
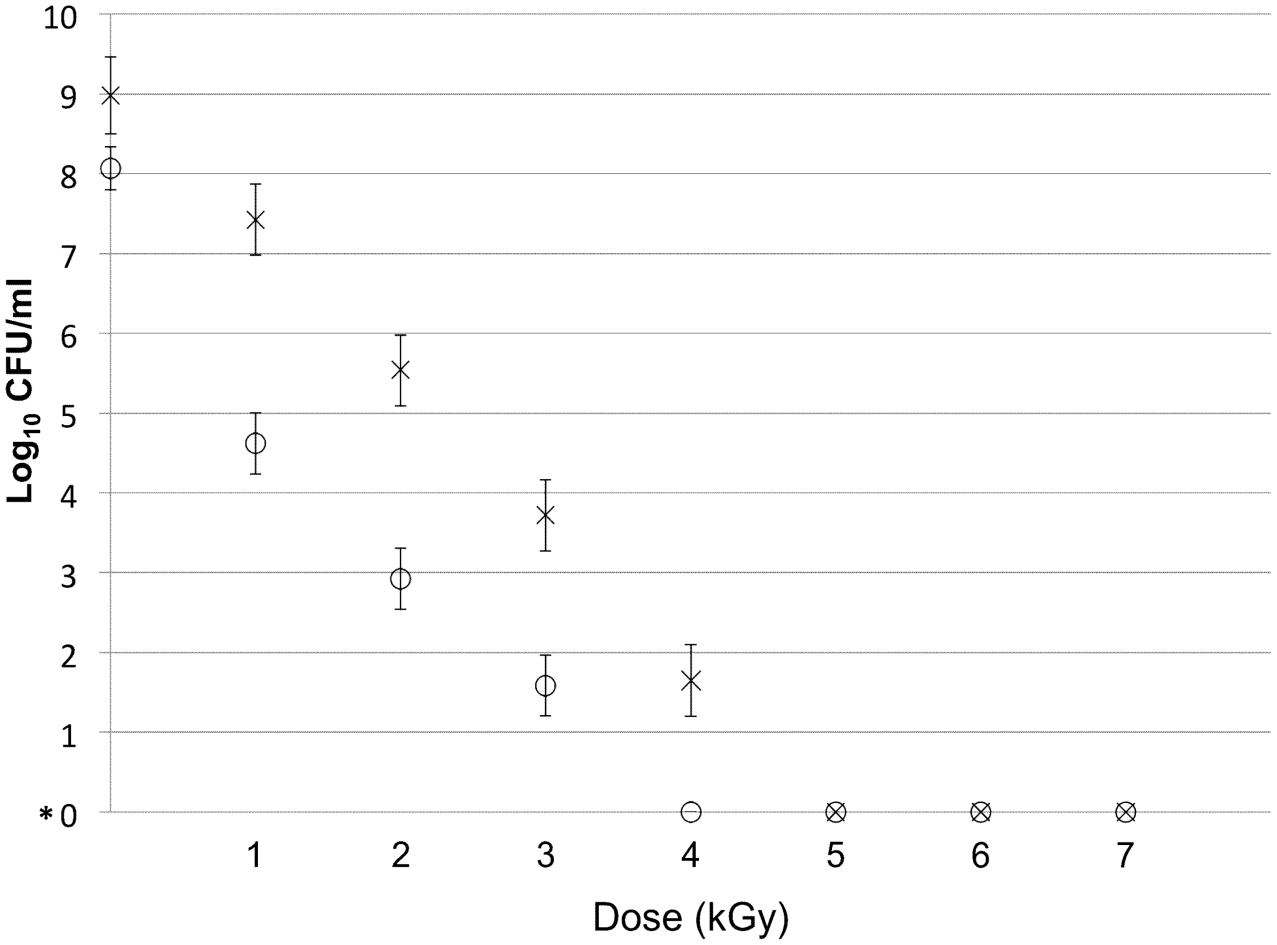
Survival curves for *R. equi* samples in 0.9% NaCl irradiated with eBeam doses ranging from 0 to 7 kGy. Survival curve for Concentration 1 (1×10^8^ CFU/ml) is indicated by the symbol ○; Survival curve for Concentration 2 (1×10^9^ CFU/ml) is indicated by the symbol ×; *0 represents true 0 and not 10^0^ = 1.

The doses required to prevent replication of *R. equi* at concentrations of 1×10^8^ and 1×10^9^ CFU/ml were 4 and 5 kGy, respectively (Fig. 1). The D_10_-values estimated for *R. equi* strain 5–331 in 0.9% NaCl exposed to 10-MeV, 18-kW eBeam irradiation for the 2 concentrations were similar (0.48 [0.37 to 0.69] and 0.53 [0.47 to 0.61]); approximately 0.505 kGy) and did not differ significantly (P>0.05).

The green/red fluorescence ratio increased significantly (P<0.05) with the percentage of intact bacteria in all groups except the heat-inactivated group (Fig. S1A, B, C and D), indicating that eBeam irradiation did not damage bacterial membrane integrity but that heat-inactivation did. Using TEM, the overall integrity of the outer bacterial cell wall of all treated groups was preserved (Fig. S2A, B, C, and D). Changes affecting the wall were confined to the layered cell wall (LCW) of all groups, and were more severe among bacteria of heat-inactivated groups suggesting a more severe compromise of the cell wall integrity (consistent with the fluorescent-based results). The internal cell contents were only morphologically affected in bacteria of the heat-inactivated group. Noticeable changes included enlarged nuclear areas that were admixed with a filamentous material and inconspicuous glycogen-like deposits (Fig. S2D).

### Systemic immune response

#### Cell-mediated immune response

**Figure 2:**
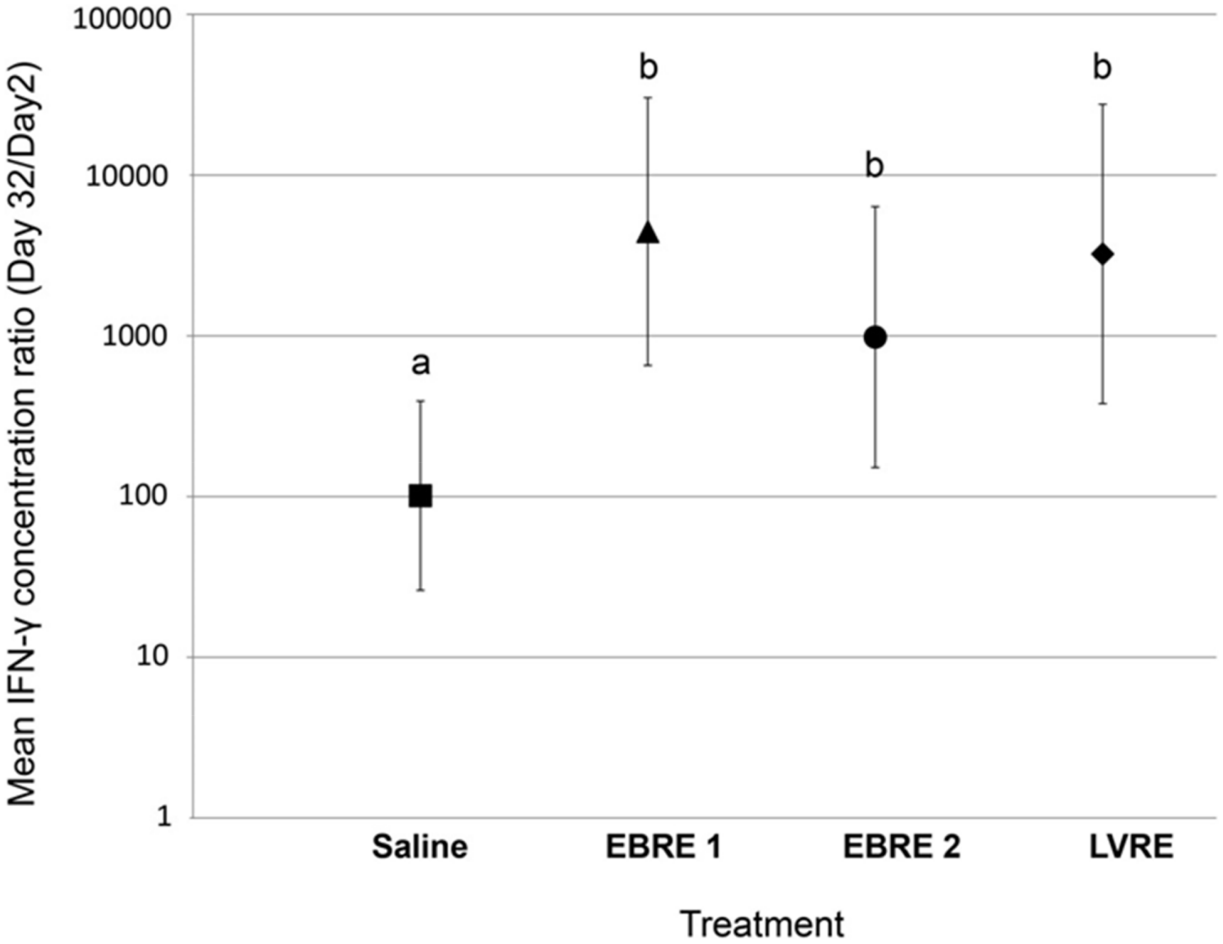
Ratio of IFN-γ concentration in culture media of eBeam inactivated *R. equi-*stimulated isolated peripheral blood mononuclear cells. Relative quantities on day 32 relative to day 2 (log_10_-transformed) from 34 foals in 4 treatment groups: 1) Saline: enteral adjuvant only controls (N = 9); 2) EBRE 1: foals receiving 1×10^11^ *R. equi* eBeam irradiated with 4 kGy enterally (N = 10); 3) EBRE 2: foals receiving 2×10^10^ *R. equi* eBeam irradiated with 5 kGy enterally (N = 9); and, 4) LVRE: foals receiving 1×10^10^ live, virulent *R. equi* enterally (N = 6). Bars with differing letters indicate significant (P<0.05) differences among groups.

Stimulation with ConA significantly (P<0.05) stimulated IFN-γ production in cultured PBMCs from foals from all treatment groups on both days 2 and 32 relative to unstimulated cells, and stimulation with eBeam irradiated *R. equi* resulted in significantly greater IFN-γ production on day 32 (P<0.05) relative to unstimulated control (media only) (Fig. S3). The IFN-γ response was significantly (P<0.05) greater for foals in the LVRE, EBRE 1, and EBRE 2 groups than for foals in the saline control group (Fig. 2).

#### Humoral immunity

Serum concentrations of total IgA, IgG_4/7_, and IgG_3/5_ decreased significantly with age for foals in all groups (Fig. S4); however, there were no significant differences in the decline of total immunoglobulins among groups.

**Figure 3:**
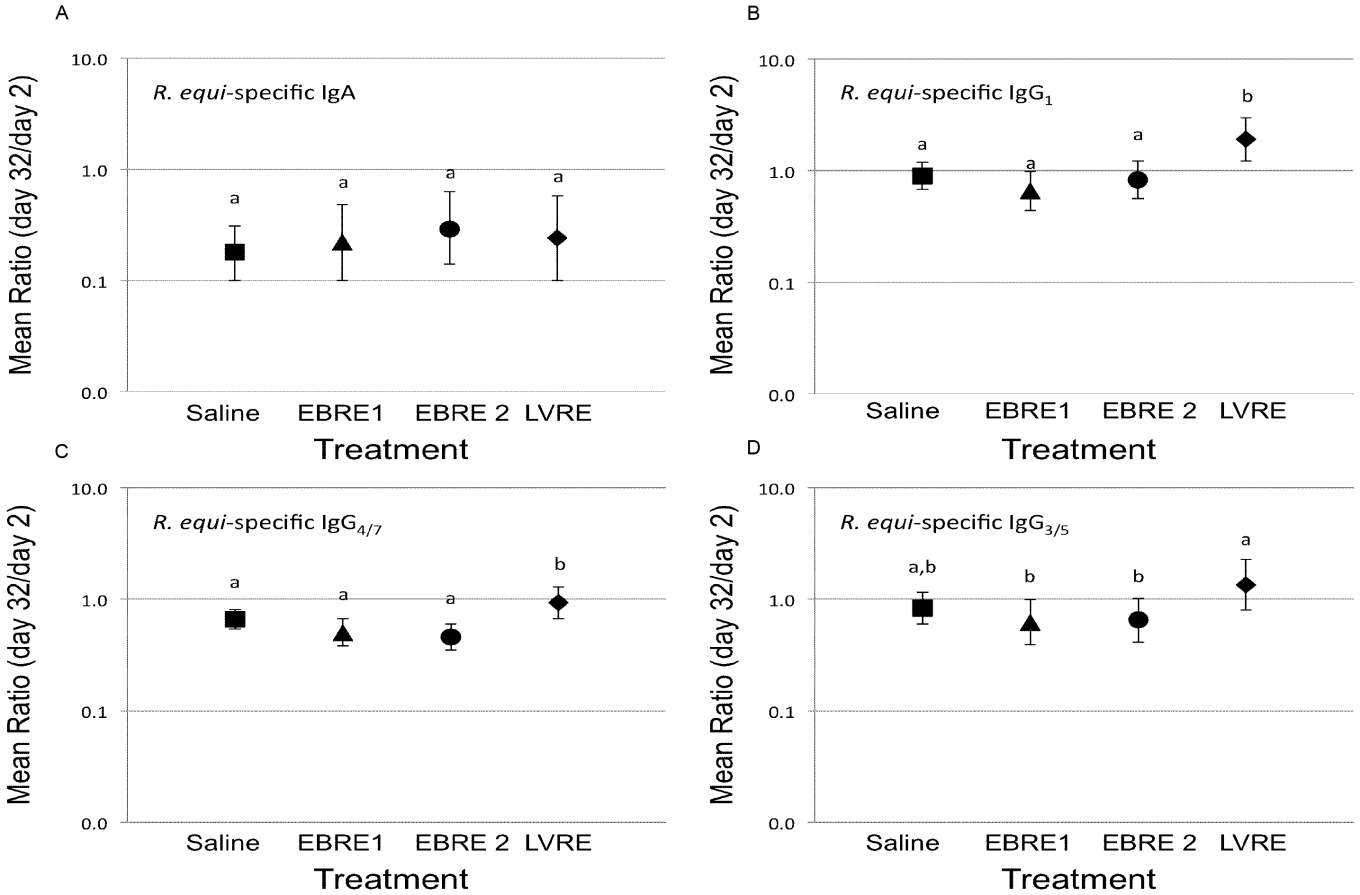
Mean Ratio of *R. equi-*specific IgA and IgG isotypes on serum samples from foals. OD on day 32 relative to day 2 (log_10_-transformed) from 34 foals in 4 treatment groups as described in Fig. 2. Bars with differing letters indicate significant (P<0.05) differences among groups. A) *R. equi*-specific IgA; B) *R. equi*-specific IgG_1_; C) *R. equi*-specific IgG_4/7_; D) *R. equi*-specific IgG_3/5_.

There were no significant differences among groups in values of the day 32-to-day 2 ratio of *R. equi*-specific serum IgA (Fig. 3A); however, the ratios were significantly (P<0.05) less than 1 for all groups. The day 32-to-day 2 ratios of serum IgG_1_ and IgG_4/7_ were significantly (P<0.05) greater for the LVRE group than other groups (Fig. 3B and C); there were no other significant differences among groups. Similarly, the ratios were significantly (P<0.05) greater for the LVRE group than the 2 vaccine groups (but not controls; Fig. 3D). Note that the magnitudes of increase were small.

### Mucosal humoral immune response

#### Naso-pharyngeal samples

**Figure 4:**
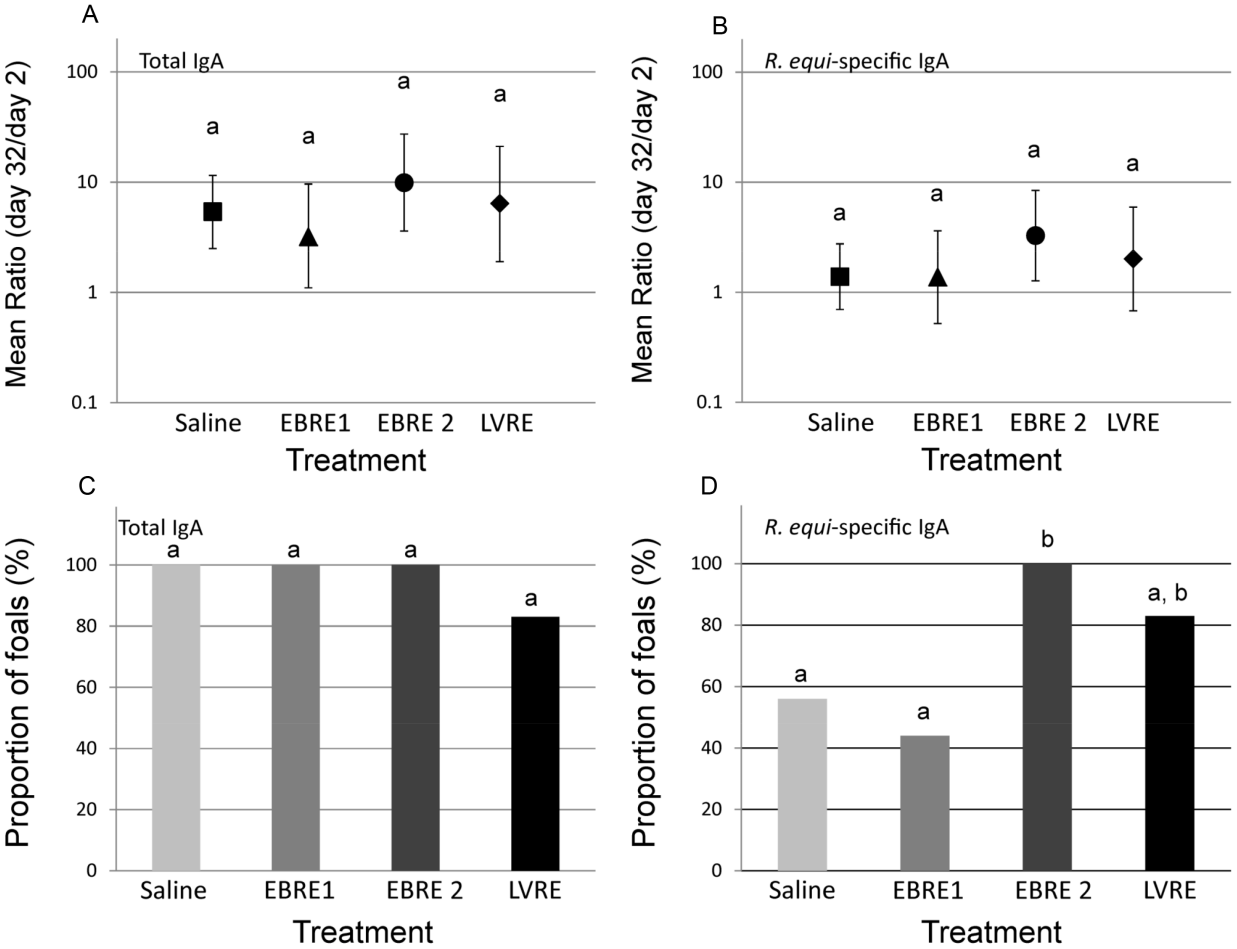
Mean ratios of total and *R. equi*-specific IgA in naso-pharyngeal samples. Relative quantities on day 32 relative to day 2 (log_10_-transformed) of IgA from 34 foals in 4 treatment groups as described in Fig. 2. Bars with differing letters indicate significant (P<0.05) differences among groups. A) Mean ratio (95% confidence interval) concentration total IgA; B) Mean ratio OD *R. equi*-specific IgA; C) Proportion of foals with increase in total IgA from day 32 relative to day 2; D) Proportion of foals with increase in *R. equi*-specific IgA from day 32 relative to day 2.

Total IgA concentration in naso-pharyngeal samples were increased for all but 1 foal; however, there were no significant differences among groups in either the relative magnitude of increase from day 2 to day 32 (Fig. 4A) or the proportion of foals that had increased IgA following vaccination (Figure 4B), although the EBRE2 and LVRE groups tended to be increased. Although the relative increase of *R. equi*-specific IgA in NP samples tended to be greater for the EBRE2 and LVRE groups (Fig. 4B), there were no significant differences among groups.

The proportions of foals that had increased *R. equi*-specific IgA, however, differed significantly among groups (P = 0.0223; Fisher's exact test; Fig. 4D). Post-hoc specific pair-wise comparisons indicated that the proportion of foals in the EBRE2 group that had increased *R. equi*-specific IgA was significantly (P<0.05) greater than that of the saline controls (Fig. 4D).

#### BAL fluid

The ratio of total and *R. equi*-specific IgA and IgG isotypes for day 32 relative to day 2 were significantly (P<0.05 for all) greater than 1 for all groups, indicating a significant increase with age (Fig. S5); however, there were no significant differences among groups in the values of these ratios. No significant association was observed in either total or *R. equi*-specific IgA between samples of BAL fluid and NP swabs from individual foals (Fig. S6).

### Maternal Influence

All mares had detectable total and *R. equi*-specific immunoglobulins of all classes in both mammary secretions and serum. There was a significant (P<0.05) correlation between mammary secretions and mare serum for total and *R. equi*-specific IgA, IgG_1_, IgG_4/7_, IgG_3/5_, except for total concentrations of IgG_1_ and IgG_4/7_; however, no differences among their respective foals treatment group were observed (data not shown).

Because mammary secretions and mare serum concentrations were correlated for most isotypes, and because we did not have access to colostrum (produced in the first 24 h PP), we studied the correlation of immunoglobulins in mammary secretions with that of foal serum. There was a significant (P<0.05) positive association for IgA and a tendency (0.05<P<0.11) for a positive association for IgG_4/7_ and IgG_3/5_ between mammary secretions and foal serum on day 2; there was no apparent association for IgG_1_ (Fig. S7). No significant differences among vaccine/treatment groups were observed. There was a significant (P<0.05) correlation between mare's mammary secretions concentration and foals NP swab concentrations for both total and *R. equi*-specific IgA on day 2 of life (data not shown); however, there was no significant difference among vaccine/treatment groups.

## Discussion

The objectives of this study were to render *R. equi* non-replicating with eBeam irradiation and to examine the immunogenicity of the eBeam irradiated *R. equi* administered intragastrically to foals. Replicability of *R. equi* was inversely related to eBeam irradiation dose (Fig. 1), as previously reported for other microorganisms including *Escherichia coli* K-12 [42], *Bacillus atrophaeus* [43], and avian influenza virus [29]. As expected, a higher dose of irradiation was required to completely inhibit replication of a higher concentration of *R. equi*. Thus, the dose of irradiation for a vaccine preparation would need to be empirically established for the target bacterial concentration. Depending on the bacterial growth phase, a higher dose may be needed for complete inhibition of bacterial replication because bacterial cells in logarithmic growth phase can have multiple copies of their genomes per cell [44].

At the doses selected, outer cell wall integrity appeared to be conserved (Fig S1 and S2). We observed similar fluorescence ratios for both concentrations of irradiated bacteria tested and live samples for all time-points (day 1, weeks 1, 2, and 4), whereas the heat-inactivated samples were considered damaged at all time-points (Fig S1). This finding was anticipated because eBeam irradiation was expected to damage the bacterial DNA [44], but damage to the cell wall might be dose-dependent and consequently titratable. For example, eBeam irradiation of spores of *Bacillus* spp. caused membrane disruption with cytoplasm leakage when high doses (i.e., ≥10.4 kGy) were used but not at lower doses [45]. We did not, however, evaluate bacterial structure in samples irradiated with > 5 kGy in our study. Using TEM (Fig S2), all control and irradiated samples preserved the overall structural integrity of the cell wall [46], meaning that changes in the cell wall indicative of destruction or perforation of the bacterial cell wall were not observed. However, morphologic changes affecting ultrastructural components of the cell wall (e.g., the layered cell wall) and internal structures (e.g., nuclear area and glycogen-like deposits) were observed. Although variation in severity of changes involving the ultrastructural components of the cell wall was found between the irradiated and heat-inactivated groups, morphologic changes in internal structures only were detected in the heat-inactivated bacteria. Preservation of the cell wall integrity after eBeam irradiation has also been shown for *Paracoccidioides brasiliensis* [47], and membrane damage of *Bacillus* spp. spores was observed only at high doses of eBeam irradiation [45]. Although the evaluation of the ultrastructure in our study did not reveal changes in the cell wall indicative of perforation or destruction of the cell in any of the treatment groups, data from the fluorescence assay, coupled with the presence of more severe ultrastructural changes affecting the layered cell wall of heat-inactivated bacteria, indicate that the cell wall of heat-inactivated bacteria were more severely compromised relative to the other treatment groups. For this study, we used a heat-inactivation protocol of 85°C for 30 min. Other heat-killing protocols using more prolonged exposure, higher temperatures, or both likely would have caused more pronounced changes in the *R. equi* ultrastructure. Overall, we observed that eBeam inactivation resulted in better maintenance of normal membrane integrity and structure than heat inactivation.

We observed that these structurally intact but non-replicating bacteria were immunogenic in neonatal foals. We chose to evaluate immune responses during the first month of life on the basis of evidence that natural infection with *R. equi* generally occurs early in life [48], [49]. We elected to use ratios (day 32 values relative to day 2) because of considerable variation in absolute values among individual foals and between ages (e.g., declining total antibody concentrations or increasing IFN-γ production with age).

Cell-mediated immune responses are of irrefutable importance to immunity to intracellular pathogens, including *R. equi* [50]. Neonatal foals, however, are known to have age-related impaired ability to produce IFN-γ [51], [52], which is essential for activating macrophages to kill intracellular *R. equi* [53]. Consistent with previous findings, we observed that IFN-γ expression by PBMCs increased with age (both basal and stimulated expression; Fig 2). More importantly, neonatal foals vaccinated with either dose of eBeam irradiated *R. equi* produced significantly greater IFN-γ in response to stimulation with *R. equi* antigens (lysate) than did controls, and this indicator of CMI responses was similar to that generated by the LVRE group (the positive control group; Fig 2 and Fig S3). These results indicate that enteral mucosal vaccination with irradiated bacteria can stimulate systemic CMI responses in neonatal foals in the face of maternal transfer of antibody (including antibodies against *R. equi*) and natural exposure to environmental *R. equi* similar to those induced by enterally-administered live *R. equi*. Thus, even immunologically naïve newborn foals can be primed to fight intracellular pathogens such as *R. equi* at an early age. The results from the LVRE group are further important because they extend our knowledge of CMI responses to enteral administration of live, virulent *R. equi*, the only approach repeatedly demonstrated to protect foals against subsequent experimental intrabronchial challenge with virulent *R. equi*. Alternatively, a non-specific immune response could be occurring *in vitro*, through stimulation of innate immune cells (such as monocytes or macrophages). Nonetheless, vaccination of foals with the eBeam inactivated *R. equi* vaccine induced an immune response that was significantly different from foals receiving saline plus adjuvant, but similar to that of the LVRE group, demonstrating the immunomodulatory effect of this vaccine in newborn foals, regardless of the cell source of the IFN-γ produced.

The concentration of total immunoglobulins of all isotypes in foal serum decreased with age but there was no significant difference among vaccine/treatment groups (Fig. 3 and Fig S4). Age-related decline in maternal antibody has been demonstrated in foals [54], [55], and was expected as a result of consumption and catabolization of maternally-derived immunoglobulin [55]. Foals from the LVRE group had significantly higher day 32-to-day 2 ratios of serum *R. equi*-specific IgG_1_ and IgG_4/7_ compared to other groups, and of IgG_3/5_ compared to both groups of vaccinates (but not saline control foals). The importance of these findings remains to be determined. Reported associations of specific IgG isotypes with protection against *R. equi* are inconclusive and conflicting. Initially, it was suggested that IgG_1_ was expected to be protective because it represented a Th1 isotype, whereas and IgG_3/5_ and IgG_4/7_ were Th2 isotypes [56], while both IgG_1_ and IgG_4/7_ interact with Fc receptors on effector cells and activate complement [57]. Subsequent studies, however, have indicated that IgG isotype dominance is not indicative of protection against *R. equi* in foals [17]. The magnitude of observed increase was modest for IgG_3/5_ and IgG_4/7_, with neither isotype for the ratio of day 32-to-day 2 being significantly greater than 1. Nonetheless, enteral administration of eBeam inactivated bacteria did not result in similar systemic antibody responses as enteral administration of live *R. equi*. The relevance of our results for increased ratios for R. equi-specific IgG_1_ and IgG_4/7_ induced in the LVRE group to protection against infection is an important consideration that remains to be determined.

Nasal mucosal *R. equi*-specific IgA appeared to be increased among foals in the higher-dose vaccine group (EBRE 2) than saline controls; the increase for the EBRE 2 foals was most similar to the LVRE group (positive controls) in magnitude of the ratio and proportion of foals whose ratio increased between days 2 and 32 (Fig 4). IgA is an important immunoglobulin at mucosal surfaces that functions primarily as a neutralizing antibody, but can also opsonize and activate complement [58]. Infection with *R. equi* in foals is thought to occur by inhalation of the bacterium [50], so IgA in nasal secretions may be an important barrier to *R. equi* infections in nasal passages by either neutralizing inhaled bacteria or by opsonizing them for subsequent phagocytosis and killing by neutrophils in the lungs. Although CTB is an adjuvant known to induce IgA responses at mucosal surfaces [59], we nonetheless observed a significantly higher proportion of foals from the EBRE2 group with increased *R. equi*-specific nasal IgA compared to the saline control group. We observed that both total and *R. equi*-specific nasal IgA amounts increased significantly with age, consistent with what has been reported previously for total IgA [49]; to our knowledge, this is the first such report for *R. equi*-specific nasal IgA. These findings indicate nasal mucosal immunity against *R. equi* may be relatively diminished in newborn foals, possibly rendering them more susceptible infection. Our findings differ from a study of *Streptococcus equi* subspecies *equi*, in which an adequate passive transfer of *S. equi*-specific antibodies was observed soon after colostrum intake [60]. The reasons for this discrepancy between studies is unclear, but might be attributable to differences in the host-agent interaction of the 2 organisms, differences in background exposure and vaccination of mares, and differences between studies in methods for evaluating antibody concentrations.

Because *R. equi* primarily causes pneumonia in foals, we evaluated immunoglobulin concentrations in BAL fluid of study foals (Fig S5 and S6). Similar to IgA from NP samples, we observed age-related increases (i.e., day 32-to-day 2 ratios significantly > 1) in BAL fluid concentrations of total and *R. equi*-specific IgA, IgG_1_, IgG_3/5_, and IgG_4/7_; however, no significant differences were observed among vaccine/treatment groups for any isotypes. As was observed in the foals of this study, IgG and not IgA is the most abundant antibody in human BAL fluid [61]. The concentrations of *R. equi-*specific immunoglobulins of all isotypes in BAL fluid were very low, and in some instances undetectable, especially on day 2. We thus repeated analyses after concentrating the BAL fluid but results were essentially identical to those presented for the original analysis (data not shown). It is unclear to what extent our BAL technique affected our results. We chose to use a low-volume BAL (30 ml) on the basis of previous research in sheep, in an attempt to yield more concentrated BAL fluid [62]–[64]. Larger volumes (such as 180 ml [65] or 500 ml [14]) are used to obtain BAL samples from foals. Conceivably, a larger lavage volume might have provided greater contact with lung tissue or yielded more immunoglobulins following concentration of a larger volume.

Both total and *R. equi*-specific antibodies of all isotypes were identified in both mammary secretions and serum from mares. It has been shown in mares that the concentration of IgG antibodies in mammary secretion decrease rapidly postpartum [66], and we recognize that mammary secretion samples from day 2 PP are likely different than colostrum ingested during the first 24 hours PP; nevertheless, there were positive associations between all immunoglobulin isotypes in the dams' mammary secretions day 2 PP and foals serum and NP swab samples (Fig S7). One frequent concern regarding vaccination of newborns is the presence and interference of maternal antibodies that could neutralize the vaccine, impairing the newborn's response to the vaccine. We did not observe this in the present study; in fact, we observed the generation of a CMI response in spite of the presumed presence of maternal antibodies. This situation has also been demonstrated with vaccination against measles in 6-month-old infants [67], where there was significant generation of IFN-γ producing CD4^+^ T cells in spite of the presence of maternal antibodies, demonstrating that mucosal vaccination in neonates can be efficacious in priming the immune system.

Our study has a number of limitations. First, intra-gastric administration of 4 doses of vaccine is impractical for large-scale use at farms. Arguably, it might be considered less cumbersome, labor-intensive, and risky for foals than the accepted and widespread use of transfusion of hyperimmune plasma to foals to reduce the incidence of *R. equi* pneumonia [5]. A second limitation is that we determined the IFN-γ concentration using supernatant of cultured PBMCs stimulated *in vitro* with either ConA or eBeam inactivated *R. equi.* We are aware that different populations of cultured cells could be producing IFN-γ; nevertheless, we were able to demonstrate that vaccinated foals responded better than saline control foals to eBeam inactivated *R. equi*, irrespective of the cell source, and in a similar way to foals receiving LVRE, known for inducing protective immune responses against *R. equi* challenge. Another limitation is that we did not evaluate efficacy of the vaccine. We also need to point out that we have not optimized the vaccine dose nor have we optimized the choice and the concentration of adjuvants. We recognize that no vaccine is proven efficacious until protecting foals against disease from the pathogen of interest. On the basis of our evidence that the vaccine is indeed immunogenic, we have initiated challenge studies of this candidate vaccine.

In summary, we demonstrate that eBeam can safely inactivate *R. equi*, without compromising the cell wall integrity, for potential use in vaccines. We also demonstrate that *R. equi* inactivated with eBeam doses of either 4 or 5 kGy can be immunogenic in foals when administered enterally with CTB as adjuvant.

## Supporting Information

Figure S1 **Ratio of green/red fluorescence using the fluorescence-based LIVE/DEAD BacLight bacterial viability kit for Concentration 1 (approximately 1×10^8^ colony-forming CFU/ml; square) and Concentration 2 (approximately 1×10^9^ CFU/ml; triangle) eBeam irradiated, live (diamond shape) and heat-inactivated samples (circle).** A) Day 1, B) Week 1, C) Week 2, and D) Week 4 of storage at 4°C.
(TIF)

Figure S2 **Ultrastructure of live, heat-inactivated, and eBeam irradiated** ***R. equi.*** **a) Live group, week 4.** Normal morphologic appearance of the nuclear area (NA). b) Concentration 2 eBeam irradiated, week 4. Similar morphologic appearance of the NA compared to the live bacterium of image “a”. The arrows are indicating invaginations of the layered cell wall. c) Heat-inactivated group, day 1. Nuclear area (NA) markedly vacuolated and has increased electron lucency. d) Live group, day 1. Closer magnification of a live bacterium depicting the localization of the layered cell wall (black arrows) and of glycogen-like material (white arrowheads). e) Concentration 2 eBeam irradiated with 5 kGy, day 1. Closer view of radiated bacteria demonstrating intact layered cell walls (black arrowheads), invaginations of the layered wall (arrow), and preservation of glycogen-like material (white arrowheads). f) Heat-inactivated, day 1. Closer magnification of a heat-killed bacterium that presents large areas where the layered cell wall is either not present (arrows) or presents marked invagination/coiling (arrowheads). Note the vacuolated nuclear area (*), and inconspicuous glycogen-like material. Concentration 2 (b) and Live bacteria (a) remains intact after 4 weeks of refrigeration, whereas heat-inactivated (c) bacteria denote changes after 12 h of refrigeration.
(TIF)

Figure S3 **Effects of stimulus (ConA, Concavalin A 5 ug/ml; Control, saline [unstimulated control]; and, eBeam irradiated** ***R. equi*** **[MOI 1∶10]) on concentration of IFN- γ in cell culture supernatant of foals at ages 2 days (panel A) or 32 days (panel B), from all treatment groups combined.** At both ages, concavalin A stimulated a significant increase in IFN- γ concentration (pg/ml). Within a panel, differing letters indicate significant differences stimuli. Between panels, different numbers indicate differences between ages.
(TIF)

Figure S4 **Mean Ratio of total IgA and IgG isotypes concentration from foal serum.** Concentration on day 32 relative to day 2 (log_10_-transformed) from 34 foals in 4 treatment groups: 1) Saline: enteral adjuvant only controls (N = 9); 2) EBRE 1: foals receiving 1×10^11^ *R. equi* eBeam irradiated with 4 kGy enterally (N = 10); 3) EBRE 2: foals receiving 2×10^10^ *R. equi* eBeam irradiated with 5 kGy enterally (N = 9); and, 4) LVRE: foals receiving 1×10^10^ live, virulent *R. equi* enterally (N = 6); Bars with differing letters indicate significant (P<0.05) differences among groups. A) Total IgA; B) Total IgG_1_; C) Total IgG_4/7_; D) Total IgG_3/5_.
(TIF)

Figure S5 **Mean Ratio of total and** ***R. equi*** **-specific IgA and IgG isotypes on BAL fluid from foals.** Relative quantities concentrations (total) and OD (*R. equi*-specific) on day 32 relative to day 2 (log_10_-transformed) from 34 foals in 4 treatment groups as described in Fig. S4. Bars with differing letters indicate significant (P<0.05) differences among groups. A) Total IgA; B) Total IgG_1_; C) Total IgG_3/5_; D) Total IgG_4/7_. E) *R. equi*-specific IgA; F) *R. equi*-specific IgG_1_; G) *R. equi*-specific IgG_3/5_; H) *R. equi*-specific IgG_4/7_.
(TIF)

Figure S6 **Association between Mean Ratio** ***R. equi-*** **specific IgA concentration from foal NP swab eluates and BAL fluid.** Relative quantities on day 32 relative to day 2 (log_10_-transformed) from 34 foals in 4 treatment groups as described in Fig. S4. There was no significant association (P = 0.5907; Pearson's correlation coefficient  = 0.0956) between the BALF *R. equi-*specific IgA and the nasal *R. equi-*specific IgA values for foals.
(TIF)

Figure S7 **Association between mammary secretions and foal serum samples on day 2 for** ***R. equi*** **-specific immunoglobulins.** A) IgA; the association was weak but statistically significant (P<0.0001); B) IgG_1_; the association was weak and not statistically significant (P = 0.1345); C) IgG4/7; the association was weak but statistically significant (P<0.0001); D) IgG3/5; the association was weak but statistically significant (P<0.0001).
(TIF)
