# Supplementary figures and images for: Immunogenicity of an Electron Beam Inactivated Rhodococcus equi Vaccine in Neonatal Foals
Source: PLoS One. 2014 Aug 25;9(8):e105367. doi: 10.1371/journal.pone.0105367 (PMC4143214; doi:10.1371/journal.pone.0105367)

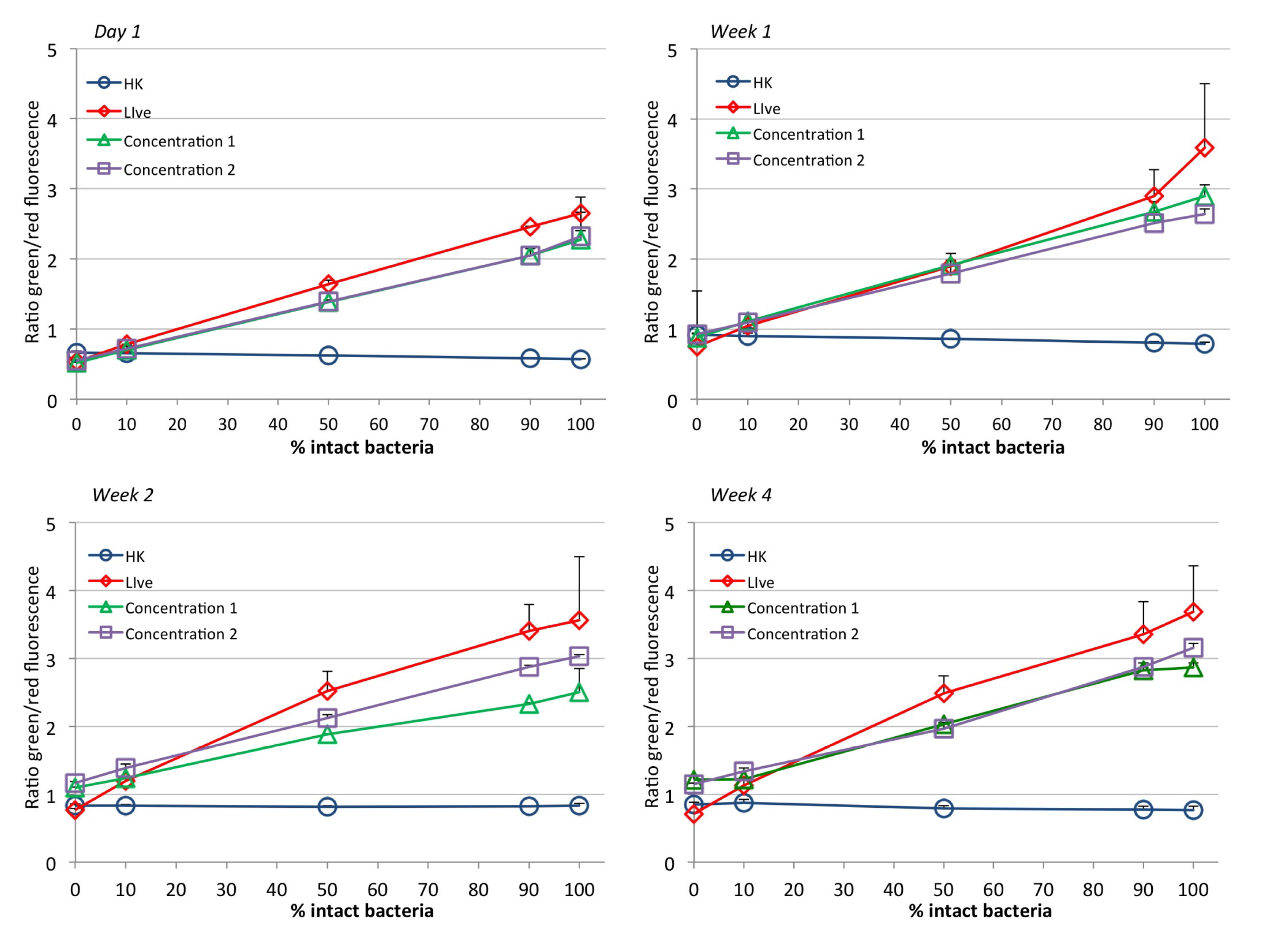

Supplement: Figure S1 — Ratio of green/red fluorescence using the fluorescence-based LIVE/DEAD BacLight bacterial viability kit for Concentration 1 (approximately 1×10^8^ colony-forming CFU/ml; square) and Concentration 2 (approximately 1×10^9^ CFU/ml; triangle) eBeam irradiated, live (diamond shape) and heat-inactivated samples (circle). A) Day 1, B) Week 1, C) Week 2, and D) Week 4 of storage at 4°C. (TIF) [file pone.0105367.s001.tif]

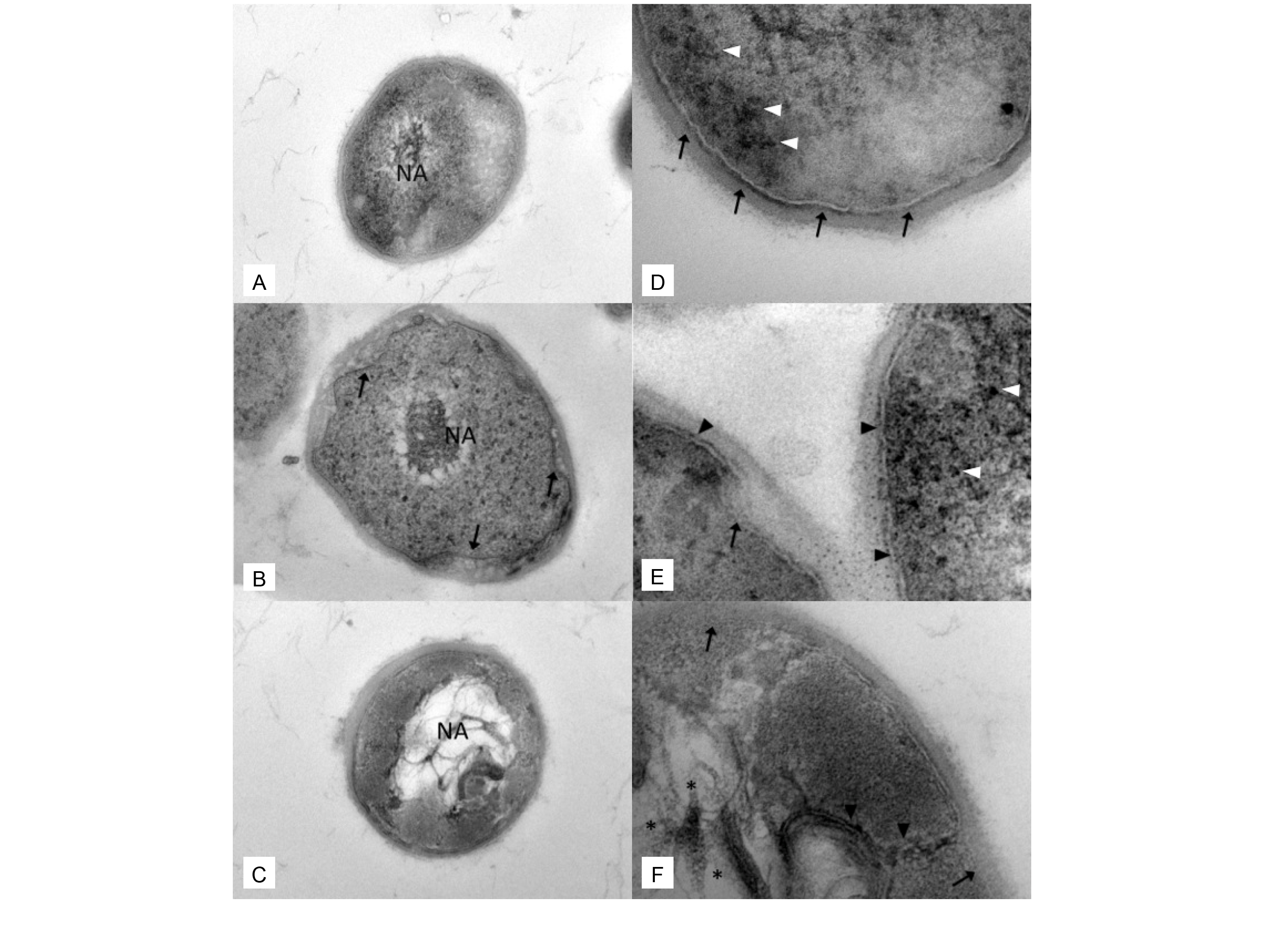

Supplement: Figure S2 — Ultrastructure of live, heat-inactivated, and eBeam irradiated R. equi. a) Live group, week 4. Normal morphologic appearance of the nuclear area (NA). b) Concentration 2 eBeam irradiated, week 4. Similar morphologic appearance of the NA compared to the live bacterium of image “a”. The arrows are indicating invaginations of the layered cell wall. c) Heat-inactivated group, day 1. Nuclear area (NA) markedly vacuolated and has increased electron lucency. d) Live group, day 1. Closer magnification of a live bacterium depicting the localization of the layered cell wall (black arrows) and of glycogen-like material (white arrowheads). e) Concentration 2 eBeam irradiated with 5 kGy, day 1. Closer view of radiated bacteria demonstrating intact layered cell walls (black arrowheads), invaginations of the layered wall (arrow), and preservation of glycogen-like material (white arrowheads). f) Heat-inactivated, day 1. Closer magnification of a heat-killed bacterium that presents large areas where the layered cell wall is either not present (arrows) or presents marked invagination/coiling (arrowheads). Note the vacuolated nuclear area (*), and inconspicuous glycogen-like material. Concentration 2 (b) and Live bacteria (a) remains intact after 4 weeks of refrigeration, whereas heat-inactivated (c) bacteria denote changes after 12 h of refrigeration. (TIF) [file pone.0105367.s002.tif]

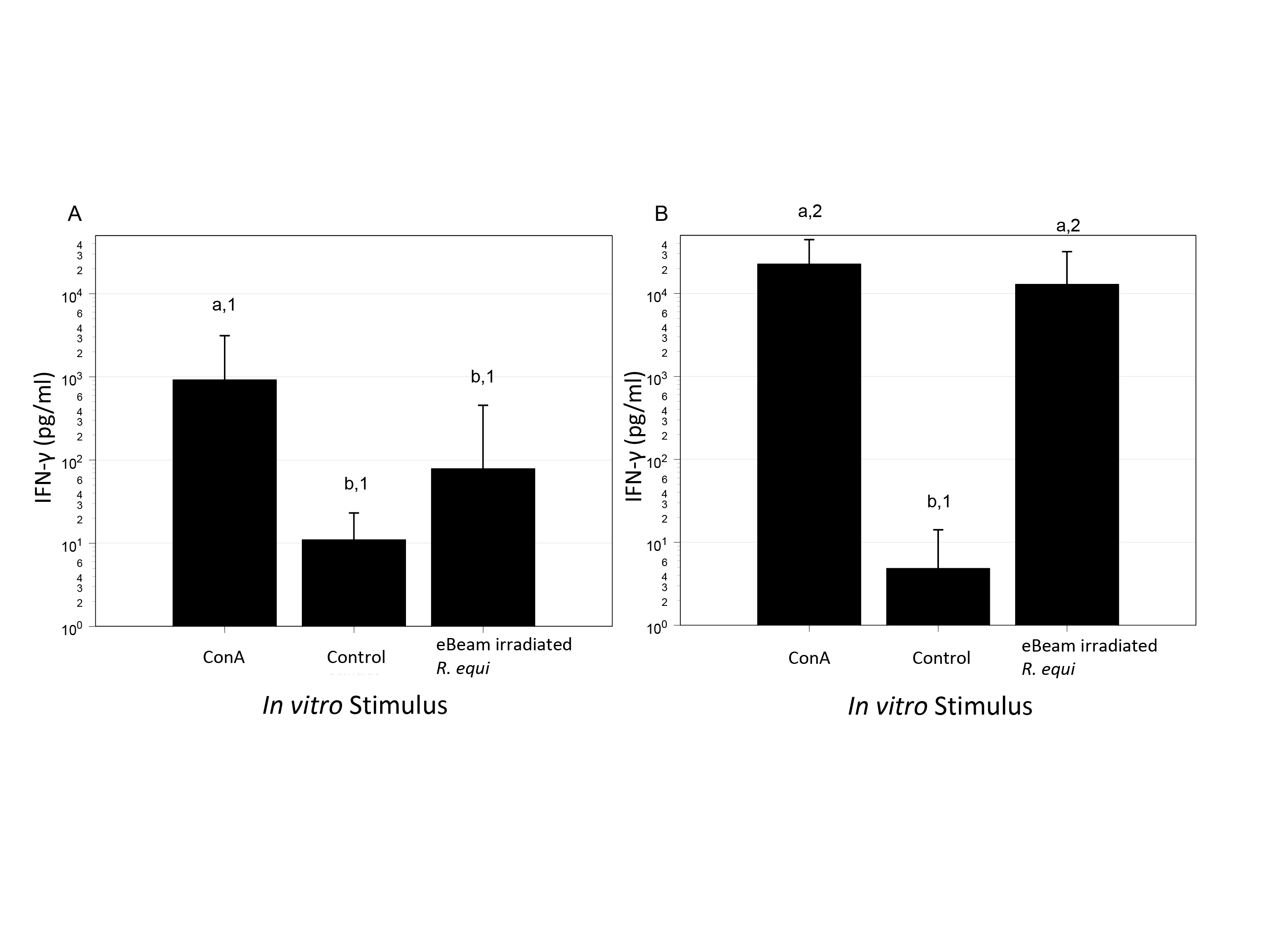

Supplement: Figure S3 — Effects of stimulus (ConA, Concavalin A 5 ug/ml; Control, saline [unstimulated control]; and, eBeam irradiated R. equi [MOI 1∶10]) on concentration of IFN- γ in cell culture supernatant of foals at ages 2 days (panel A) or 32 days (panel B), from all treatment groups combined. At both ages, concavalin A stimulated a significant increase in IFN- γ concentration (pg/ml). Within a panel, differing letters indicate significant differences stimuli. Between panels, different numbers indicate differences between ages. (TIF) [file pone.0105367.s003.tif]

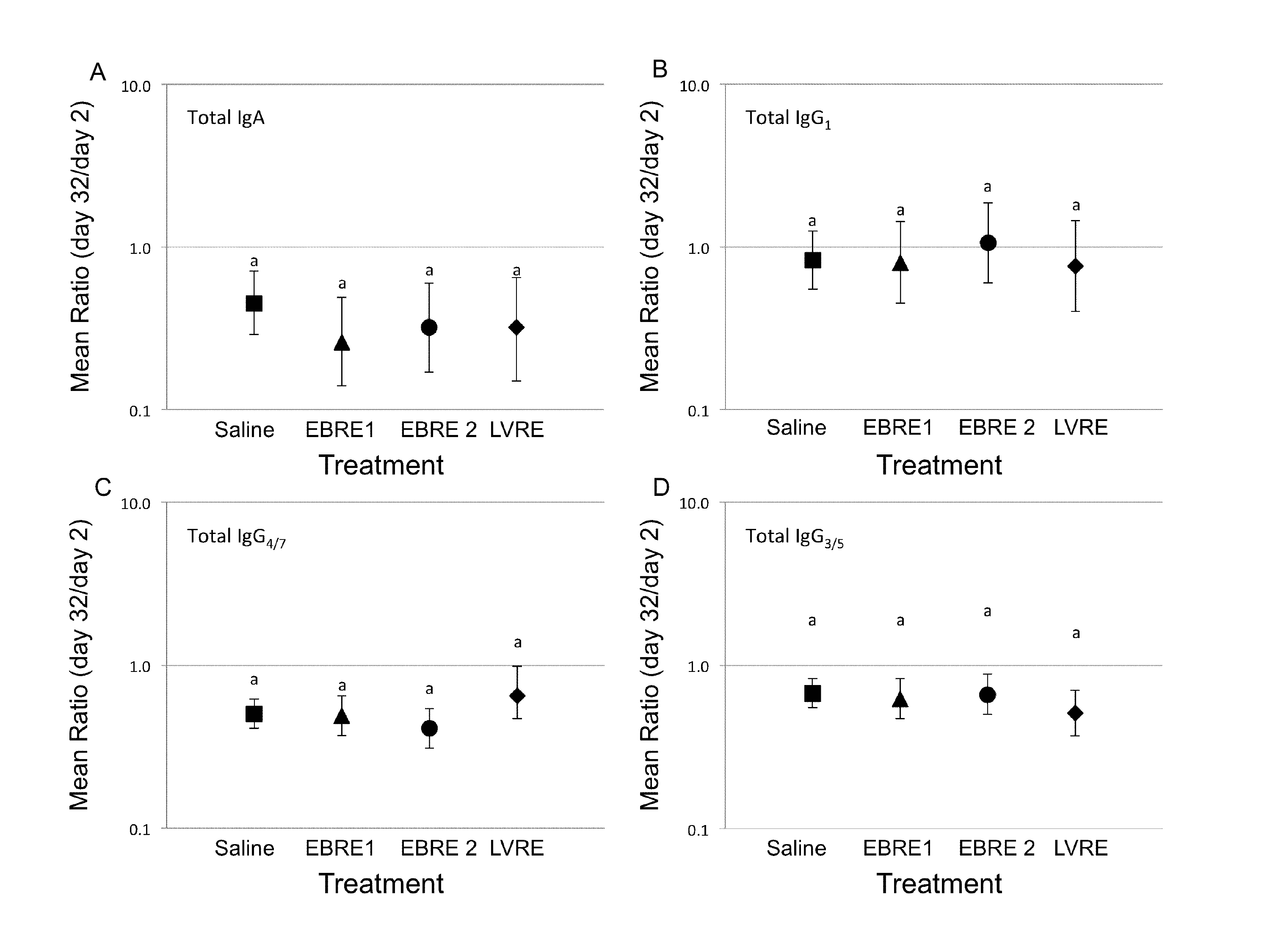

Supplement: Figure S4 — Mean Ratio of total IgA and IgG isotypes concentration from foal serum. Concentration on day 32 relative to day 2 (log10-transformed) from 34 foals in 4 treatment groups: 1) Saline: enteral adjuvant only controls (N = 9); 2) EBRE 1: foals receiving 1×10^11^ R. equi eBeam irradiated with 4 kGy enterally (N = 10); 3) EBRE 2: foals receiving 2×10^10^ R. equi eBeam irradiated with 5 kGy enterally (N = 9); and, 4) LVRE: foals receiving 1×10^10^ live, virulent R. equi enterally (N = 6); Bars with differing letters indicate significant (P<0.05) differences among groups. A) Total IgA; B) Total IgG1; C) Total IgG4/7; D) Total IgG3/5. (TIF) [file pone.0105367.s004.tif]

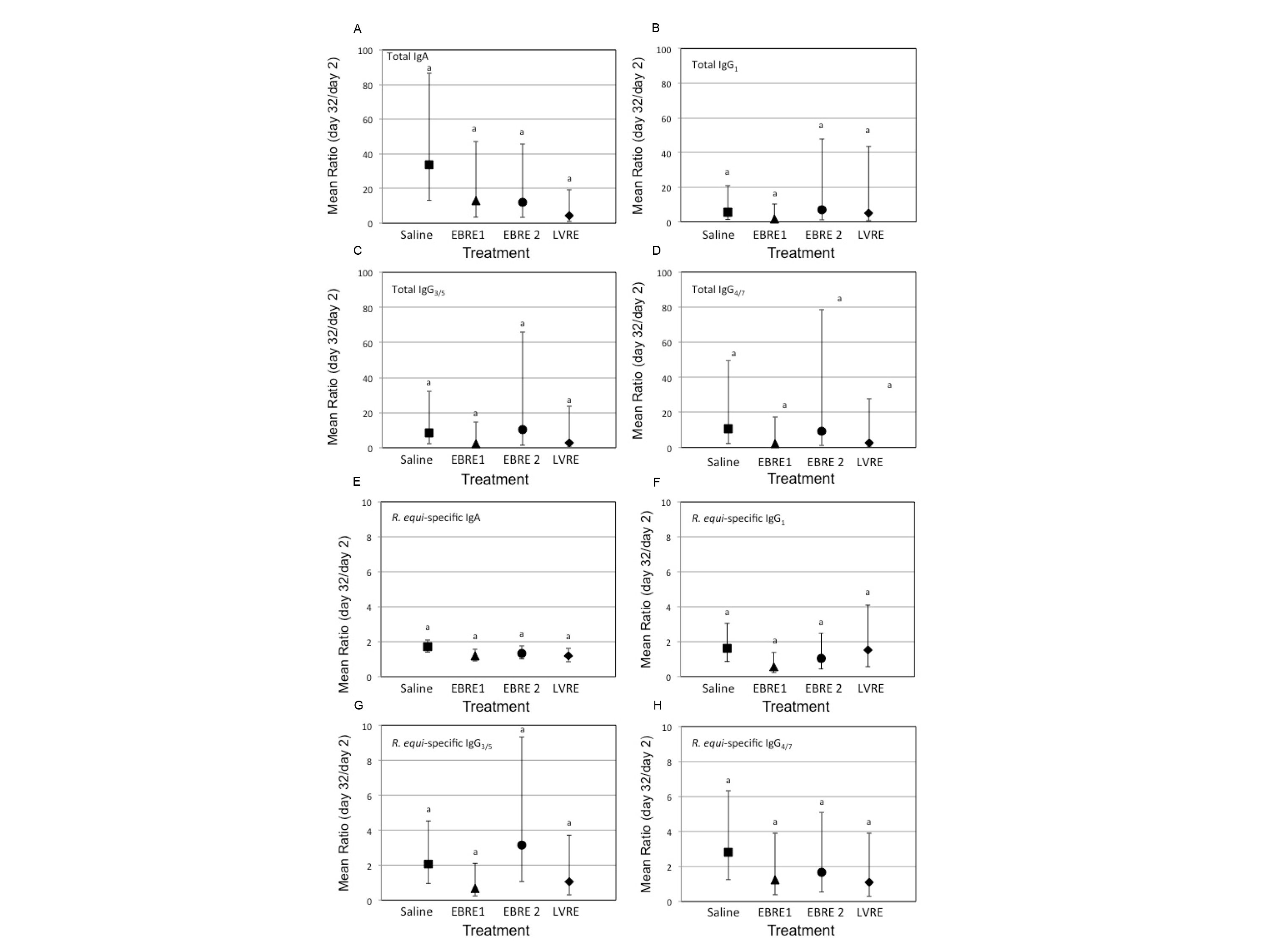

Supplement: Figure S5 — Mean Ratio of total and R. equi -specific IgA and IgG isotypes on BAL fluid from foals. Relative quantities concentrations (total) and OD (R. equi-specific) on day 32 relative to day 2 (log10-transformed) from 34 foals in 4 treatment groups as described in Fig. S4. Bars with differing letters indicate significant (P<0.05) differences among groups. A) Total IgA; B) Total IgG1; C) Total IgG3/5; D) Total IgG4/7. E) R. equi-specific IgA; F) R. equi-specific IgG1; G) R. equi-specific IgG3/5; H) R. equi-specific IgG4/7. (TIF) [file pone.0105367.s005.tif]

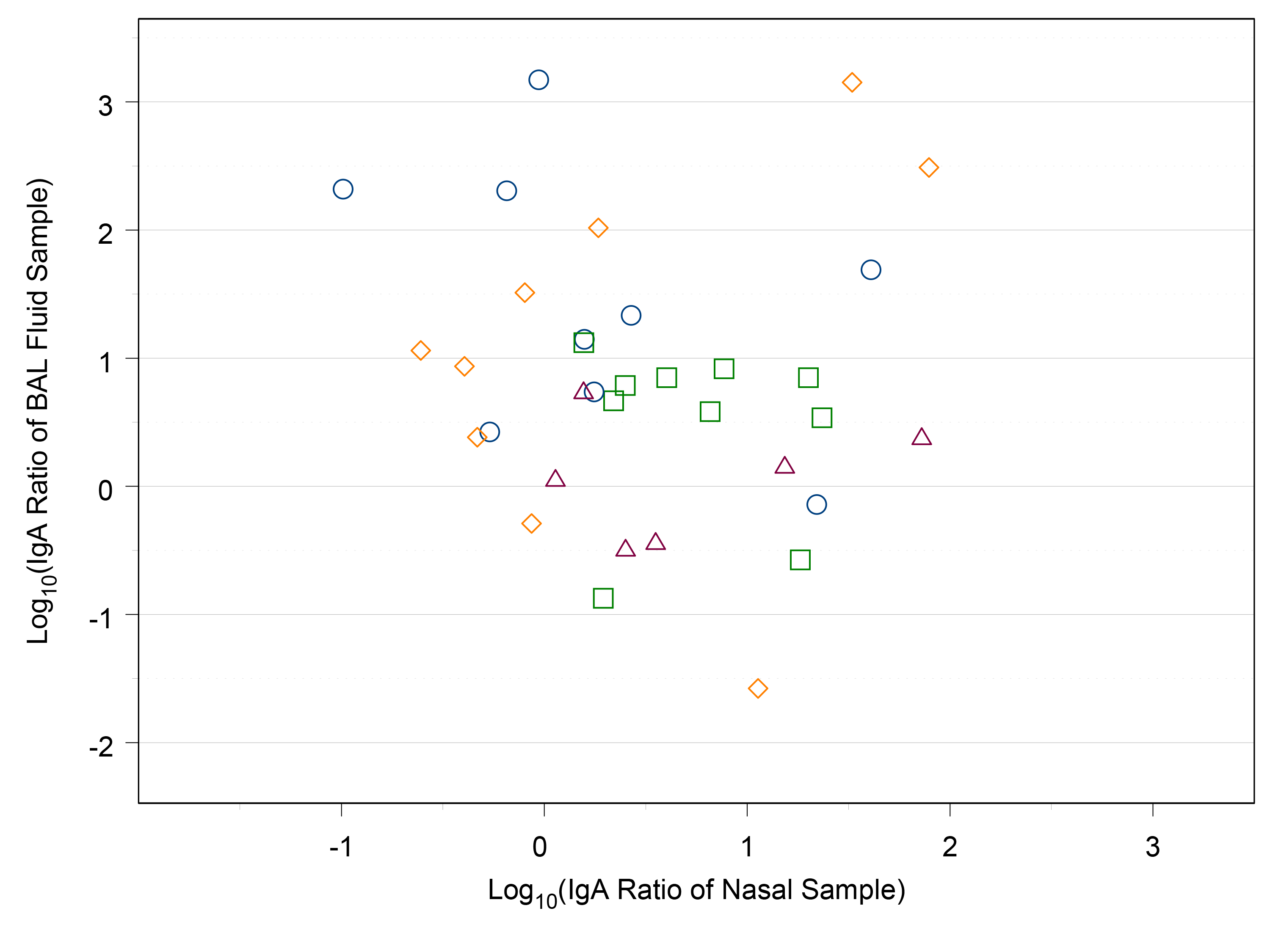

Supplement: Figure S6 — Association between Mean Ratio R. equi- specific IgA concentration from foal NP swab eluates and BAL fluid. Relative quantities on day 32 relative to day 2 (log10-transformed) from 34 foals in 4 treatment groups as described in Fig. S4. There was no significant association (P = 0.5907; Pearson's correlation coefficient = 0.0956) between the BALF R. equi-specific IgA and the nasal R. equi-specific IgA values for foals. (TIF) [file pone.0105367.s006.tif]

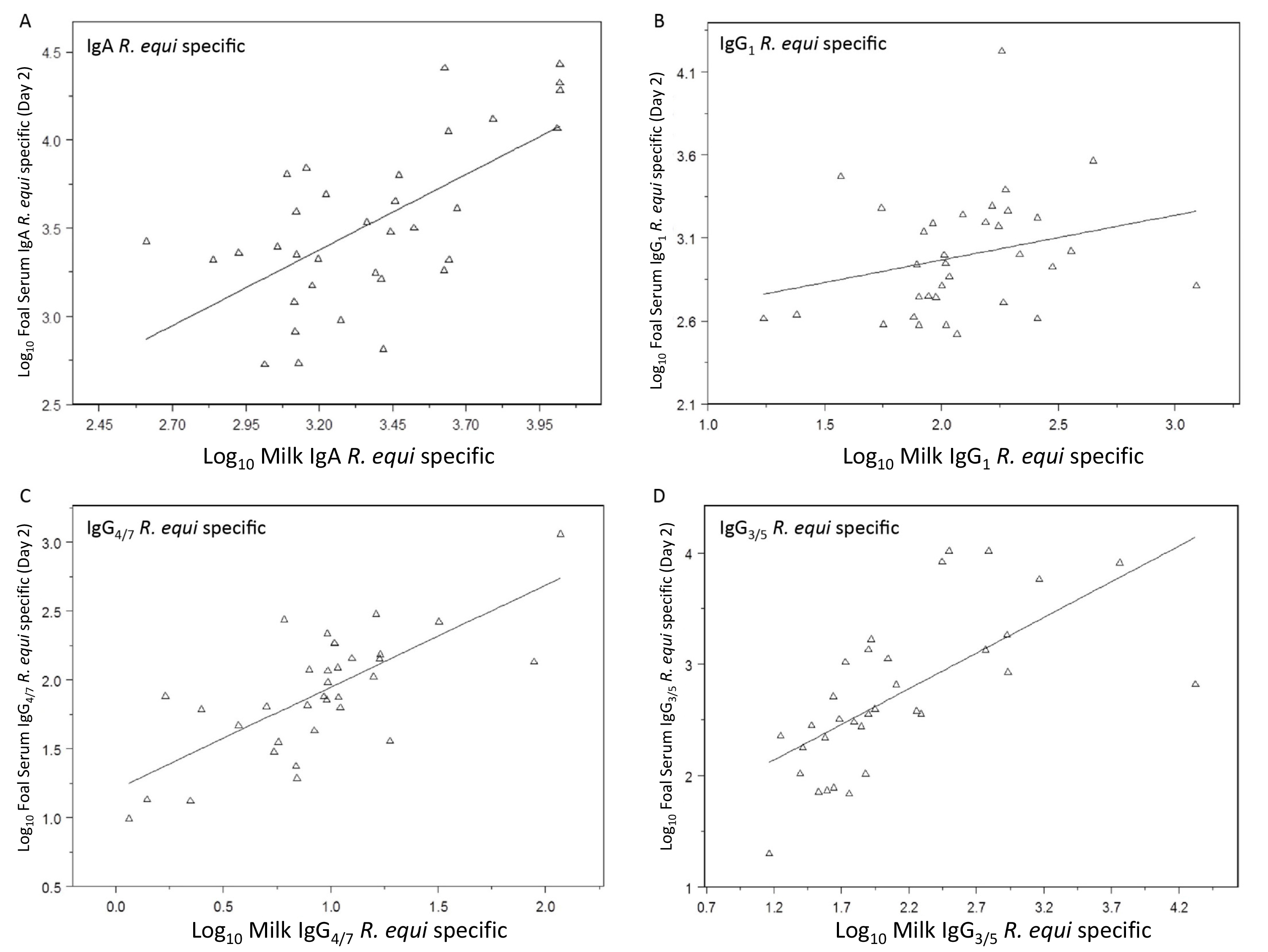

Supplement: Figure S7 — Association between mammary secretions and foal serum samples on day 2 for R. equi -specific immunoglobulins. A) IgA; the association was weak but statistically significant (P<0.0001); B) IgG1; the association was weak and not statistically significant (P = 0.1345); C) IgG4/7; the association was weak but statistically significant (P<0.0001); D) IgG3/5; the association was weak but statistically significant (P<0.0001). (TIF) [file pone.0105367.s007.tif]
